# Dual Antimicrobial Effect of Medium-Chain Fatty Acids against an Italian Multidrug Resistant *Brachyspira hyodysenteriae* Strain

**DOI:** 10.3390/microorganisms10020301

**Published:** 2022-01-27

**Authors:** Giulia Giovagnoni, Benedetta Tugnoli, Andrea Piva, Ester Grilli

**Affiliations:** 1 DIMEVET, Dipartimento di Scienze Mediche Veterinarie, Università di Bologna, Via Tolara di Sopra 50, Ozzano dell’Emilia, 40064 Bologna, Italy; giulia.giovagnoni4@unibo.it (G.G.); andrea.piva@unibo.it (A.P.); 2 Vetagro S.p.A., Via Porro 2, 42124 Reggio Emilia, Italy; benedetta.tugnoli@vetagro.com; 3 Vetagro Inc., 17 East Monroe Street, Suite #179, Chicago, IL 60603, USA

**Keywords:** swine dysentery, *Brachyspira hyodysenteriae*, multidrug resistance, antibiotics, medium chain fatty acids, antimicrobial activity, virulence genes, feed additives

## Abstract

The fastidious nature of *Brachyspira hyodysenteriae* limits an accurate in vitro pre-screening of conventionally used antibiotics and other candidate alternative antimicrobials. This results in a non-judicious use of antibiotics, leading to an exponential increase of the antibiotic resistance issue and a slowdown in the research for new molecules that might stop this serious phenomenon. In this study we tested four antibiotics (tylosin, lincomycin, doxycycline, and tiamulin) and medium-chain fatty acids (MCFA; hexanoic, octanoic, decanoic, and dodecanoic acid) against an Italian field strain of *B. hyodysenteriae* and the ATCC 27164 strain as reference. We determined the minimal inhibitory concentrations of these substances, underlining the multidrug resistance pattern of the field strain and, on the contrary, a consistent and stable inhibitory effect of the tested MCFA against both strains. Then, sub-inhibitory concentrations of antibiotics and MCFA were examined in modulating a panel of *B. hyodysenteriae* virulence genes (*tlyA*, *tlyB*, *bhlp16*, *bhlp29.7*, and *bhmp39f*). Results of gene expression analysis were variable, with up- and downregulations not properly correlated with particular substances or target genes. Decanoic and dodecanoic acid with their direct and indirect antimicrobial property were the most effective among MCFA, suggesting them as good candidates for subsequent in vivo trials.

## 1. Introduction

Swine dysentery is an economically important disease that affects fattening pigs, causing low mortality rate but a decline in growth performance and feed conversion. Animals can either show a mucohemorragic diarrhea with colitis, or no clinical signs. This condition complicates the diagnosis of swine dysentery, which already difficult due to the nature of the etiological agent: *Brachyspira hyodysenteriae* is a Gram-negative fastidious anaerobic spirochaete, problematic to in vitro isolate and cultivated from feces or infected colonic tissues of ill pigs [1]. Severe disease is caused by strongly beta-hemolytic strains, whereas weakly-hemolytic ones are usually avirulent and thus associated to less acute illness [2]. Since cultivation of *B. hyodysenteriae* is difficult, many research areas are still lacking, although hemolysis has been recognized as one of the main virulence factors. The expression of *tly* genes has been associated to the hemolytic phenotype of the strain [3]. In particular, *tlyA*, *tlyB*, and *tlyC* encode for hemolysin A, a caseinolytic protease (Clp), and hemolysin C respectively [4,5,6]. Other *Brachyspira* virulence factors are identified as the group of the outer membrane proteins that regulate the interactions with the host’s epithelial intestinal cells as well as the immune evasion although in a not yet defined mechanism [7].

Other than growth performance decline, the main economic loss due to the onset of swine dysentery is represented by the cost of prevention and treatment of the disease, since no valid vaccines are currently available [1]. The most used antibiotics in treating swine dysentery are macrolides, lincosamides, pleuromutilins, and tetracyclines [8]. In Italy, recommended antibiotics are lincomycin, tiamulin, and valnemulin, avoiding the use of macrolides for which resistance is raising exponentially [9]. Nowadays, multidrug resistance strains are spreading all over the world, making antibiotics frequently ineffective [8]. Therefore, the research of alternative antimicrobial molecules is of great importance for the control of *B. hyodysenteriae*. The main struggle is the lack of in vitro pre-screening of commonly used antibiotics and alternative molecules due to the fastidious nature of this microorganism. Medium-chain fatty acids (MCFA), particularly saturated fatty acids with a chain length of 6–12 carbon atoms, are known to be good antimicrobials, as well as gut health and growth promoters when used as feed additives in pig production [10,11,12]. For this reason, their role in control *Brachyspira hyodysenteriae* could be promising and their in vitro pre-screening is fundamental for further studies.

The aim of this study was to evaluate, in vitro, the antimicrobial power of conventional antibiotics for *B. hyodysenteriae* and MCFA against a field strain isolated in northern Italy, including the ATCC 27164 strain as reference. Moreover, a possible modulation of a panel of *Brachyspira* virulence genes has been then investigated using sub-inhibitory concentrations of these substances.

## 2. Materials and Methods

### 2.1. Bacterial Strains and Culture Conditions

The ATCC strain *Brachyspira hyodysenteriae* 27164 and a strongly-hemolytic field strain isolated in northern Italy in January 2020 from a symptomatic pig were used in this study. The strains were stored at −80 °C and, when thawed, were maintained on Tryptone Soya Agar with 5% Sheep Blood (Oxoid, Basingstoke, UK) for up to 4 days in anaerobic jars (Oxoid, Basingstoke, UK) with AnaeroGen™ sachets (Oxoid, Basingstoke, UK) at 37 °C.

### 2.2. Chemicals and Test Solutions

Antibiotics and MCFA were purchased from Alfa Aesar (Thermo Fisher GmbH, Kandel, Germany). Stock solutions of hexanoic acid, octanoic acid, decanoic acid, dodecanoic acid, and doxycycline were prepared in 70% (*v*/*v*) ethanol at 800 mM (MCFA) and 2560 μg/mL (doxycycline), so that the final and maximum concentration of ethanol tested in the agar and broth dilution method was 2.2% and 0.55% for MCFA, respectively, and 1.75% for doxycycline in both the tests. The remaining antibiotics (tylosin, lincomycin, and tiamulin) were prepared at 256 μg/mL in Tryptone Soya Broth (TSB; Oxoid, Basingstoke, UK) at pH 7 or in ATCC Medium 1827 (Brain Heart Infusion broth supplemented with heat inactivated fetal bovine serum and glucose) at pH 6.5 for agar dilution and broth dilution method, respectively. All solutions were filter-sterilized, then stored at +4 °C and brought back to room temperature before each use.

### 2.3. Antimicrobial Susceptibility Testing

The antimicrobial activity of the tested compounds was determined using agar dilution method and broth dilution method in 48-well microtiter plates. Testing conditions were performed following the guidelines of Clinical and Laboratory Standards Institute (CLSI) [13].

#### 2.3.1. Agar Dilution Method

Tryptone Soya Agar plates were prepared in triplicate by adding 5% (*v*/*v*) defibrinated horse blood (Oxoid, Basingstoke, UK) and 14% (*v*/*v*) of serial two-fold dilutions of antibiotics or MCFA. Antibiotics were tested at final concentrations ranging from 64 to 0.008 μg/mL, whereas MCFA from 25 to 0.39 mM. Control plates with TSB or ethanol at 2.2% (depending on the stock solution used) were made to control the growth of the strains. All the plates were inoculated with one of the two *B. hyodysenteriae* strains as follows: 4-day cultures of either the ATCC or the field strain on agar were harvested and resuspended in TSB up to the turbidity corresponding to McFarland #1. The bacterial suspension was then diluted in order to reach 10^6^ CFU/mL. With this suspension, every plate was inoculated with seven drops of 20 μL each.

After 4 days of anaerobic incubation at 37 °C, Minimal Inhibitory Concentrations (MIC) was established. MIC was defined as the lowest concentration of antimicrobial substance able to prevent the hemolysis of blood agar plates.

#### 2.3.2. Broth Dilution Method

For the broth dilution test in 48-well microtiter plates, two-fold dilutions of the substances were prepared in ATCC Medium 1827. Antibiotics were tested at final concentrations ranging from 64 to 0.008 μg/mL, whereas in a separate plate MCFA from 6.25 to 0.20 mM. Every concentration was tested in triplicate. Control wells with TSB or ethanol at 1.75% (depending on the stock solution used) were made to control the growth of the strains.

Again, the inoculum was prepared for the agar dilution method to reach a final inoculum of 10^6^ CFU/well in a final volume of 500 μL/well.

After 4 days of anaerobic incubation at 37 °C on a shaker (150 rpm), the MIC value was defined as the lowest concentration that resulted in null absorbance (630 nm) registered with Varioskan™ LUX Multimode Microplate Reader (Thermo fisher Scientific Inc., Waltham, MA, USA). Sub-inhibitory doses were chosen for the following virulence gene expression study. More precisely, the doses below the detected MIC (half MIC doses) or the highest concentration tested (if no MIC was found) were selected.

### 2.4. Virulence Gene Expression Study

Gene expression analysis was performed on samples of *B. hyodysenteriae* strains cultured with sub-inhibitory doses of antibiotics or MCFA from the broth dilution method. In brief, the content of each selected well (500 μL) was harvested, centrifuged for 5 min at 5000× *g*, and the supernatants were discarded. The pellets were resuspended in 100 µL of TE buffer supplemented with 1 mg/mL of lysozyme and incubated for 10 min at 37 °C. The RNA extraction and its conversion in cDNA were performed according to Giovagnoni et al. [14]. *B. hyodysenteriae* virulence gene expression was determined with a real-time PCR according to Giovagnoni et al. [14], testing the genes listed in Table 1.

mRNA expression was normalized using *gyrB* and *rpoD* as housekeeping genes. After determining the threshold cycle (Ct) for each gene, the relative changes in mRNA expression of *B. hyodysenteriae* strains grown with antibiotics or MCFA compared to controls were calculated using the 2^−ΔΔCt^ method [15].

### 2.5. Statistical Analysis

The data were analyzed with GraphPad Prism v. 9.2.0 (GraphPad Software, Inc., San Diego, CA, USA), and differences were considered significant at *p* ≤ 0.05. For gene expression data, *t* test was performed in order to compare every substance with the control.

## 3. Results

### 3.1. Antimicrobial Susceptibility Testing

MIC values of antibiotics and MCFA obtained by agar and broth dilution methods are summarized in Table 2 for both the ATCC and the field strain.

### 3.2. Gene Expression Study

**Figure 1:**
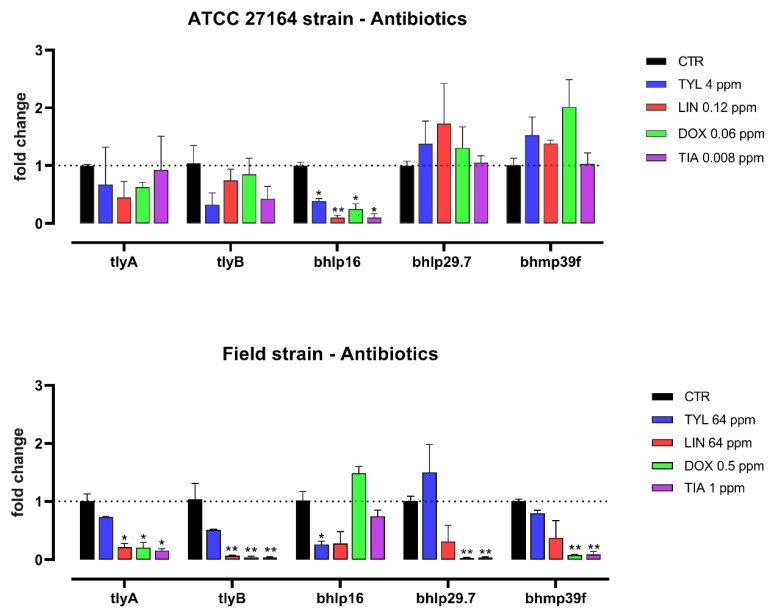
mRNA expression of *tlyA*, *tlyB*, *bhlp16*, *bhlp29.7*, and *bhmp39f* for *B. hyodysenteriae* ATCC 27164 and the field strain cultured alone (CTR), or with sub-inhibitory doses of antibiotics. Data are presented as mean (n = 3) ± SEM. For each gene, significant differences between each substance and its control are marked by asterisks (* for *p* < 0.05 and ** for *p* < 0.01). TYL = tylosin, LIN = lincomycin, DOX = doxycycline, TIA = tiamulin.

**Figure 2:**
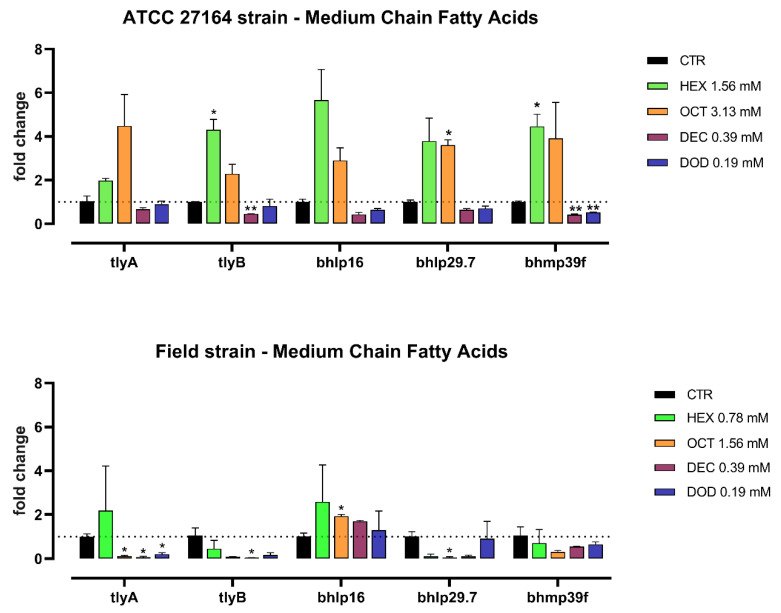
mRNA expression of *tlyA*, *tlyB*, *bhlp16*, *bhlp29.7*, and *bhmp39f* for *B. hyodysenteriae* ATCC 27164 and the field strain cultured alone (CTR), or with sub-inhibitory doses of medium-chain fatty acids. Data are presented as mean (n = 3) ± SEM. For each gene, significant differences between each substance and its control are marked by asterisks (* for *p* < 0.05 and ** for *p* < 0.01). HEX = hexanoic acid, OCT = octanoic acid, DEC = decanoic acid, DOD = dodecanoic acid.

The expression of *Brachyspira* virulence genes is represented in Figure 1 and Figure 2 for antibiotics and MCFA, respectively.

The sub-inhibitory doses of the tested substances have variously regulated the expression of hemolysin-associated genes and outer membrane proteins genes. Tylosin significantly decreased the expression of *bhlp16* for both the strains. Lincomycin downregulated *bhlp16* in ATCC strain and both the hemolysin-associated genes (*tlyA*, *tlyB*) in the field strain (*p* < 0.05), showing a trend for lower *bhlp16* level (*p* = 0.10). For the field strain, *tlyA*, *tlyB*, *bhlp29.7*, and *bhmp39f* were downregulated by doxycycline, which also reduced *bhlp16* mRNA level in the ATCC strain. Tiamulin significantly decreased the expression of *bhlp16* in ATCC strain and *tlyA*, *tlyB*, *bhlp29.7*, and *bhmp39f* in the field strain.

Hexanoic acid significantly upregulated the level of *tlyB* and *bhmp39f* in the ATCC strain. In the field strain, octanoic acid downregulated *tlyA* and upregulated *bhlp16*, whereas the same acid increased the expression of *bhlp29.7* in the ATCC strain. Decanoic acid was able to decrease the level of *tlyB* and *bhmp39f* in the ATCC strain (*p* < 0.01), and the level of *tlyA* and *tlyB* in the field strain (*p* < 0.05). Finally, *bhmp39f* was downregulated in the ATCC strain by dodecanoic acid (*p* < 0.01), which also decreased the expression of *tlyA* in the field strain (*p* < 0.05).

## 4. Discussion

*B. hyodysenteriae* is a fastidious bacterium, able to grow only in particular and enriched conditions. For this reason, antimicrobial susceptibility tests are difficult to carry out and, without a proper in vitro preliminary screening, a possible misuse of antibiotics and a consequent spread of antibiotic resistant bacteria are likely to occur. Moreover, information about in vitro antimicrobial activity of bioactive compounds as alternative to antibiotics is still scarce. This can be considered a major problem dealing with non-antibiotic treatment of swine dysentery, since the possibility of in vitro pre-screening these molecules could considerably limit the large number of in vivo trials.

During the previous years, numerous studies worldwide assessed the antimicrobial susceptibility of various *B. hyodysenteriae* strains, as recently reviewed by Hampson et al. [8]. These studies were carried out even using commercially available panels trying to establish a standardized and unique protocol [16,17]. This effort stems from the need to obtain antimicrobial susceptibility testing data before treating animals, since numerous cases of antibiotic resistance have been recorded. In fact, only in 2017 the Clinical and Laboratory Standards Institute (CLSI) provided guidance for antimicrobial agent susceptibility testing and breakpoints for fastidious bacteria for veterinary use, including *B. hyodysenteriae* [13].

In this study, for both the ATCC and the field strain, agar and broth dilution methods were found to be quite comparable, with MIC values being found with broth generally lower than those of agar dilution method, as already documented [18,19,20]. A panel of antibiotics including tylosin, lincomycin, doxycycline, and tiamulin was tested because of their wide use in treatment of swine dysentery [21,22]. All of these antibiotics are protein synthesis inhibitors by targeting the 50S (tylosin, lincomycin, and tiamulin) or 30S (doxycycline) ribosomal unit. Universal breakpoints are suggested by the CLSI only for tylosin and tiamulin [13]. Resistance breakpoints of tylosin and tiamulin for the broth microdilution method are identified as ≥128 μg/mL and ≥1 μg/mL, respectively, indicating that in our study the ATCC strain showed susceptibility (MIC_TYL_ = 8 μg/mL; MIC_TIA_ = 0.016 μg/mL) and the field strain showed resistance (MIC_TYL_ ≥ 64 μg/mL; MIC_TIA_ = 2 μg/mL) to both antibiotics. For tiamulin, the result was confirmed by the agar dilution method with an outcome MIC of 0.125 μg/mL for the ATCC strain and 4 μg/mL for field strain, with the CLSI resistance cutoff being at ≥2 μg/mL. No official breakpoints for lincomycin and doxycycline are available. To fill the gap of lacking breakpoints and standardize the antimicrobial susceptibility tests of *B. hyodysenteriae*, cutoff values for six antibiotics were proposed by Pringle et al. [23]: even based on these assumptions, in this study the MIC of the ATCC strain always fell below these agreed values, while the MIC of the field strain fell above. The *B. hyodysenteriae* ATCC 27164 was proposed by Pringle et al. [19] as a suitable quality control strain for antimicrobial susceptibility tests, although its lower MIC for pleuromutilins pushed the research of new reference strains for tiamulin and valnemulin [17,18]. Accordingly with previous studies, our data showed reproducible results about the ATCC 27164 strain for both the broth and the agar dilution method [16,17,19,20,24,25,26]. Considering the MIC values of the field strain instead, we can assume that it can be considered a multidrug-resistant strain of *B. hyodysenteriae* isolated in the North of Italy. Unfortunately, there is limited data about antibiotic resistance in *B. hyodysenteriae* from Italy. Rugna et al. [27] reported a reduction of susceptibility to tiamulin from 2003 to 2012, whereas De Luca et al. [28] described the multidrug resistance of 10 Italian *B. hyodysenteriae* strains to tylosin, lincomycin, tiamulin, and for some also to doxycycline. In particular, decreased susceptibility of Italian strains to tylosin and lincomycin was confirmed by the presence of A2058T mutation and the acquisition of the transposon associated-*lnu*(C) gene [28], already correlated with resistance patterns to macrolides and lincosamides [29,30,31,32]. According to the Italian situation, in the majority of European studies that investigated the resistance to tylosin, lincomycin, doxycycline, and tiamulin in the last decade, it is evident that the reduction in sensitivity to both tylosin and lincomycin is common and frequently concomitant. On the other hand, resistance to tiamulin and/or doxycycline is not always observed among European strains, although, when present, it covers a high percentage of the tested strains [23,31,33,34,35,36]. This can be explained by a selective environmental pressure that results in an increasing multidrug resistance, as happens in our field strain. Clearly, antibiotic resistance is less evident for the ATCC 27164 strain because of its sampling, dating back to 1972. New effective antimicrobial alternatives can be sourced among molecules not influenced by environmental pressure.

Organic acids are widely used as feed additives in swine farming due to their dual use as antimicrobials and growth promoters in the dose range of 0.2–3% [10,11,12]. Depending on their acid dissociation constant, undissociated organic acids can pass through the bacterial membrane and, in response to the release of H^+^ and RCOO^−^ ions, are able to exert their antimicrobial action: on one hand, bacteria waste energy to restore intracellular pH and, furthermore, the anion has a toxic effect by targeting replication and metabolic functions [37]. In particular, antibacterial properties of MCFA are recognized as the most effective ones against Gram-positive bacteria and, to a lesser extent, against Gram-negative bacteria [38]. MCFA antibacterial activity against *B. hyodysenteriae* was investigated by Vande Maele et al. using broth dilution method [39]. From their findings, the bacterial response to these acids varied depending on the pH conditions and the length of the carbon chain [39]. The longer is the carbon chain and the lower is the pH, the stronger is the antimicrobial activity, with dodecanoic acid being the most powerful antimicrobial against three strains of *B. hyodysenteriae*, including the ATCC 27164 [39]. Consistently, we did observe the same differences comparing the agar dilution method (pH 7) and the broth dilution method (pH 6.5). This behavior for MCFA can be explained by the anion model theory, according to which the inhibitory effect of organic acids is highly related to their acid dissociation constant and undissociated form that in turn depends on the environmental pH [37,40]. Decanoic and dodecanoic acid showed the lowest MIC, also confirming the longer the chain, the more effective the antibacterial properties of MCFA are, the only exception being hexanoic acid with a lower MIC compared to octanoic acid, as already shown for other Gram-negative bacterial species, like *Escherichia coli* [41]. Despite the difference in relation to pH and the carbon chain length, in our study, MIC values of MCFA were more consistent between the two strains compared to the tested antibiotics. In fact, resistance development to organic acids is much less frequent and problematic than for antibiotics [10], because the bacterial susceptibility to MCFA is an intrinsic property, meaning that the bioactive power of these molecules meets a broad spectrum of microorganisms with lower probability of failure.

To our knowledge, this is the first study investigating the role of antibiotics and MCFA in modulating *B. hyodysenteriae* virulence gene expression. Targeting virulence represents a novel and alternative approach to find new antimicrobials and diminish the selective pressure of resistance issue: the target of interest is not the killing or the inhibition of pathogens, but the reduction of virulence factors required to cause disease thanks to sub-lethal or sub-inhibitory doses of antimicrobials [42]. We investigated the regulation of two categories of virulence genes correlated with an increase of severity of swine dysentery [43]: hemolysis-associated (*tlyA* and *tlyB*) and outer membrane proteins genes (*bhlp16*, *bhlp29.7*, *bhmp39f*). TlyA and TlyB are considered a pore-forming hemolysin and a caseinolytic protease (Clp), respectively [4,5]: their role in modulating the hemolytic phenotype and the virulence has been investigated, although the genetic background is not yet fully understood. *TlyA* negative *B. hyodysenteriae* mutants showed reduced hemolysis in vitro and less severe lesions in pigs and mice [44,45]. On the other side, the specific functions of *bhlp16*, *bhlp29.7*, and *bhmp39f* are unknown, but they are presumably involved in the interaction with epithelial cells. Indeed Gömmel et al. postulated that the in vitro attachment of *B. hyodysenteriae* strain B204 to IPEC-J2 cells can be driven by the action of these proteins [46].

In our multidrug resistant strain, the regulation of virulence genes appeared to be different for tylosin and lincomycin. The strain was strongly resistant to both antibiotics, consistently with the common genetic basis of the resistance to macrolides and lincosamides already reported in literature [29]. However, when treated with high but not inhibitory doses of the two antibiotics, the hemolysis-associated markers were significantly reduced by lincomycin but not by tylosin, which instead reduced only one outer membrane protein. The exact mode of action behind this dual effect is not clear and needs to be further investigated, but is likely not merely related to the resistance traits. Our field strain has been considered resistant also to doxycycline and tiamulin, although both antibiotics showed an inhibitory action in the MIC tests. In this case, low doses of both drugs below the MIC values were able to downregulate the expression of four out of five markers of virulence analyzed. Therefore, it can be suggested that doxycycline and tiamulin are more likely to positively modulate the bacterial virulence, even at sub-inhibitory doses compared to drugs towards which a strong bacterial resistance is established (tylosin and lincomycin). Similarly, it has been shown that the expression of *E. coli* K88 toxins and other pathogenic factors can be reduced by the treatment with sub-inhibitory doses of colistin and doxycycline, but not affected by high concentrations of amoxicillin against which the strain is highly resistant [41].

The ATCC strain used as reference resulted to be more sensitive to antibiotics, as already discussed, and also more stable in terms of virulence modulation. Indeed, the antibiotics used at sub-MIC doses did not affect the expression of the analyzed markers, except for one outer membrane protein that was consistently reduced irrespective of the antibiotic type. This different behavior for the ATCC strain compared to the field strain could be related to the lower doses, but higher efficacy, of antibiotics against the reference strain. However, the detailed mechanism still needs to be clarified.

The effect of MCFA on virulence gene expression was quite variable for both the field strain and the ATCC strain. We did observe a general pattern of upregulation of most virulence markers in the ATCC strain with hexanoic and octanoic acid, whereas longer MCFA, such as decanoic and dodecanoic acid, did reduce hemolysin genes in the field strain. We did not expect such difference between the two strains based on the equivalent susceptibility to the antimicrobial action of MCFA found in our MIC tests. However, this can be explained by the specific toxic effect of the anionic form that each different MCFA acquires inside bacterial cells [47].

In a recent review by Jackman et al. [12] it has been reported how MCFA can be included in feed additives as mixtures with other compounds, ranging from 0.2% to 3%, with the aim to curb *Salmonella* and *Escherichia coli* infections. Comparing these inclusions with our tested concentrations, the latter are clearly lower, but could have an interesting potential to be investigated with in vivo studies, maybe with new technologies like microencapsulation that can help the delivering of small amounts of protected ingredients to target sites of the intestine [48].

## 5. Conclusions

Although with some limitations, these data suggest that, even in a multidrug-resistant strain, virulence genes can be modulated thanks to both antibiotics and MCFA in a variable manner. Decanoic and dodecanoic acid had the lowest and most stable MIC against *B. hyodysenteriae*, and their sub-inhibitory concentrations also decreased the expression of some important virulence genes correlated to a worsening of swine dysentery. It will be interesting to elucidate the mechanism of decanoic and dodecanoic acid in the in vitro modulation of hemolytic phenotype and attachment to epithelial intestinal cells, and eventually verify their potential to control swine dysentery in in vivo.

## Figures and Tables

**Table 1:** List of primers used for real-time PCR.

Gene	Function	Sequence (5′ → 3′)	Product Length (bp)	Accession Number	Reference
*tlyA*	Hemolysin A	F: AAAGGCGTTTGTAGAATTTGGAAT R: TGTCCTACATCAAGAGCATAAACTTTTT	131	MT304819.1	[3]
*tlyB*	Clp protease	F: AAGGATTCGATAAGAAGTATGGTGCTA R: TTCGGTACTCACATAATCCTCTATCTCT	79	MT304820.1	[3]
*bhlp16 (smpA)*	Outer membrane protein	F: GCAGGTGTAGAAAAGGGATTTGG R: TCTGAAGAACTTGCTCCACCTT	107	CP015910.2	This study
*bhlp29.7 (bmpB)*	Outer membrane protein	F: TGGTTTTGCTGGAGAGTCTGA R: TCTCCGTCATTCAAAGCCTGAT	132	AY706761.1	This study
*bhmp39f*	Outer membrane protein	F: AGCCTTTCGGTATTGGCGTA R: ACAGCTATTTGAACAGGAACTGC	130	AY027775.1	This study
*gyrB*	Housekeeping	F: TGCAGGCGGTACTGCTAAAG R: GCACCTACACCGCATCCTAA	159	CP015910.2	This study
*rpoD*	Housekeeping	F: AGCTTTTGCCTCTATCTGACGA R: ACAGTTTGCCGGACAGAGAA	137	CP015910.2	This study

F = forward; R = reverse.

**Table 2:** Minimal inhibitory concentrations (MIC) of antibiotics and medium chain fatty acids (MCFA) tested against the ATCC 27164 strain and the field strain using the agar and broth dilution method.

		ATCC 27164 Strain		Field Strain	
		Agar Dilution	Broth Dilution	Agar Dilution	Broth Dilution
Antibiotics	Tylosin	16 μg/mL	8 μg/mL	>64 μg/mL	>64 μg/mL
	Lincomycin	2 μg/mL	0.25 μg/mL	>64 μg/mL	>64 μg/mL
	Doxycycline	2 μg/mL	0.12 μg/mL	4 μg/mL	1 μg/mL
	Tiamulin	0.125 μg/mL	0.016 μg/mL	4 μg/mL	2 μg/mL
MCFA	Hexanoic acid	25 mM	3.13 mM	25 mM	1.56 mM
	Octanoic acid	>25 mM	6.25 mM	25 mM	3.13 mM
	Decanoic acid	3.13 mM	0.78 mM	3.13 mM	0.78 mM
	Dodecanoic acid	0.39 mM	0.39 mM	0.39 mM	0.39 mM

## Data Availability

Data available on request due to restrictions, e.g., for privacy or ethical reasons.
